# Students Advocating for Diversity in Medical Education

**DOI:** 10.15694/mep.2019.000159.2

**Published:** 2020-01-08

**Authors:** Julia L. Moss, Eliza C. Hardy, Keiko A. L. Cooley, Samantha N. Cuffe, Madeline L. Lang, Ann Blair Kennedy

**Affiliations:** 1 University of South Carolina School of Medicine Greenville

**Keywords:** Education Environment, Ethics/Attitudes, Professionalism, Medicine, Change, Diversity, Health Equity

## Abstract

This article was migrated. The article was marked as recommended.

**Problem:** Innovations within the medical education system often come from administration and leadership, in the traditional top-down approach to preparing students for the actualities of medical practice. There is a dearth of literature showing the power of students to design and advance innovations in this same arena. As incoming classes of students are increasingly more diverse, student efforts for diversity and inclusion initiatives must be explored as avenues to effect positive change within the system.

**Approach:** Medical students at the University of South Carolina School of Medicine Greenville (UofSC SOM Greenville) formed the committee known as *Student Advocates for Diversity and Inclusion* (SADI) in Fall 2017, with the goals of enhancing the curriculum, increasing the visibility of diverse peoples within the medical school and the healthcare system, and supporting the experience of these peoples.

**Outcomes:** The report herein describes the formation of the *Student Advocates for Diversity and Inclusion* and its initial steps, including the modification of curricular practices and the development of extracurricular programs.

**Conclusion:** SADI may serve as one example of the power of students to transform medical education. Other students and schools can use the committee and its successes and challenges to implement similar programs at their respective institutions, with the goal of achieving diversity and inclusion more broadly across the medical education system.

## Problem

The medical community has long grappled with the challenges of physician diversity and health disparities. In 2018, the American Medical Association reaffirmed health equity as a priority, setting policy and advocacy around accomplishing “optimal health for all” through increased physician workforce diversity, research and programming, and targeting the social determinants of health ( Robeznieks, 2019). Addressing diversity and cultural differences in medical education is imperative to ensure future physicians can competently care for the dynamic and increasingly diverse United States population. Moreover, medical students want to learn about diversity to broaden their educational experiences during medical school ( Dogra and Karnik, 2003; Guiton, Chang and Wilkerson, 2007). National student groups, such as the Student National Medical Association (SNMA) and the Latino Medical Student Association (LMSA), have driven recruitment of medical students of underrepresented races and cultures in medicine and have advocated for inclusivity and policy to address health disparities. Despite students’ calls for increased presence of diverse students and faculty, culturally focused training, and diversity awareness in medical education, there is little literature regarding the power of students to improve diversity and inclusivity within the medical education system.

Physicians, no matter their own racial or cultural background, must be competent in caring for all patients; to that end, the Liaison Committee on Medical Education (LCME) mandates cultural competence training for all medical students. The LCME, operated by the American Medical Association and the Association of American Medical Colleges, requires accredited medical schools to 1) implement non-discrimination policy to protect students and employees, 2) work towards achieving diversity among students and employees, and 3) educate students on the basic principles of culturally competent healthcare ( Liaison Committee on Medical Education, 2018). Following the 2009 implementation of these LCME accreditation standards, students enrolling in medical school have been increasingly female as well as Black and Hispanic (groups classified as Underrepresented in Medicine (UIM)). Nevertheless, even as enrollees in medical school have become increasingly more diverse, graduates of medical school remain less diverse than the general U.S. population ( Boatright *et al.*, 2018). According to the American Association of Medical Colleges ( American Association of Medical Colleges, 2016), in 2015 6% of medical school graduates were Black or African American, 5% were Hispanic or Latino; the remaining graduates included Whites at 58.8% and Asians at 19.8%. These figures are essentially unchanged since 1995 ( American Association of Medical Colleges, 2008). The recruitment of diverse applicants is trending upward, but the graduation of diverse physicians still presents an opportunity for growth as one step towards achieving health equity.

While the accrediting bodies do not mandate specific hours for cultural competence training within the medical school curriculum, they do establish that faculty must provide instruction and content in six specific domains: health disparities, community strategies, bias, cross-cultural communication, interpreter utilization, and self-reflection. Curriculum development and research within these domains provide tremendous opportunity for students to use their diverse experiences, voices, and backgrounds to contribute to the design of their own education. Furthermore, these requirements set by the accrediting bodies entrust students with the responsibility of ensuring that they are fulfilled by their institution and implemented on their campus ( Liaison Committee on Medical Education, 2018). This more active role for medical students as partners in medical education innovation is unlikely without the support of leadership and administration. It should be emphasized that providing such opportunities for students, giving them a seat at the table as it were, can create trusting relationships that benefit all stakeholders ( Healey, Flint and Harrington, 2014). These relationships may be the foundation for effecting real change, as in correcting the disconcerting figures described above.

In the following report, we describe the development and implementation of programs, initiatives, and cultural changes led by a group of students at the University of South Carolina School of Medicine Greenville (UofSC SOM Greenville), with endorsement from and mentorship of faculty, staff, and administration. Our advocacy for diversity and inclusion demonstrates the power of collective action to achieve substantial change within an institution of medical education, by implementing innovative solutions while utilizing few resources apart from the willpower of a unified student coalition and the support of a receptive school leadership.

## Approach

### Formation of the Student Advocates for Diversity and Inclusion

In Fall 2017, eight second-year students opened the discussion on diversity and inclusivity at UofSC SOM Greenville. We students, leaders of UIM student interest groups, came together with the shared appeal for promoting an inclusive student environment and establishing a comprehensive curriculum that would adequately prepare students to work with diverse populations. Following informal discussions among ourselves as well as productive meetings with leaders in the medical school and physicians at the associated healthcare system, we took action to fill the gaps we observed in the school infrastructure and curriculum.

Faculty and staff were overwhelmingly supportive upon learning of our concerns. We first requested the formation of a formally recognized student committee on diversity and inclusion. The Student Advocates for Diversity and Inclusion (SADI) was thence formed with dual aims of improving diversity within both the curriculum and the school community. SADI commenced quarterly meetings to serve as the liaison between the school administration (including professors, administrators, physicians, and Student Affairs staff) and the involved student interest groups (including Lesbian, Gay, Bisexual and Transgender (LGBT) Health, SNMA, Medical Students for Health Advocacy, and the LMSA). Our short-term goals included curricular changes and extracurricular programs to expose students to the experiences of diverse patients and providers, which would contribute to meeting the committee’s stated mission of:

“...advancing the integration of diversity and inclusion within the medical school’s curriculum; supporting those student organizations with an emphasis on diversity; and fostering an environment for meaningful discussions centered on diversity and inclusion at UofSC SOM Greenville.”

### Curricular changes

One key component of the UofSC SOM Greenville curriculum is the Integrated Practice of Medicine (IPM) course, which teaches students throughout the four years of medical school clinical skills, professionalism standards, and patient-centered care. IPM is a team-taught course that contains the majority of the behavioral, social, and population health curriculum, including cultural competence content. Course faculty continually review class sessions for areas of improvement with regards to LCME requirements and opportunities for method and content development. This review process, combined with the advocacy of SADI, yielded new class sessions on topics of diversity and expansion of the cultural competence curriculum (e.g. in the first year, Introduction to LGBTQ Populations and Social Determinants in Population Health During Pregnancy; in second year, Loss of Trust: History of Medicine, Race, and Discrimination, and a Patients with Obesity panel).

One area in which we wanted to add diversity was the clinical scenarios in the pre-clinical years. In the IPM course, faculty present students with a new case each week to evaluate and discuss. We wanted the patients in these cases to reflect the diverse patients and situations we would see in our clinical practice, with the idea that this would encourage students to begin thinking early on in their careers about the ways in which these factors impact health and patient care. In order to be more intentional about this, we created a checklist (Supplement 1) to guide case presentations to better reflect the diversity of our community. The checklists itemize various patient characteristics including race/ethnicity, sexual/gender identity, English language fluency, religion, socioeconomic status, disability, education level, and neighborhood. IPM faculty members were asked to ensure each of their case scenarios incorporated at least two characteristics from the list and to consider these characteristics in guiding student discussions about how health disparities influence the delivery of patient-centered care. The diversity checklists now guide clinical case development and classroom discussion on how diversity and discrimination create health disparities and affect health outcomes. How the cases have specifically changed since the implementation of the checklist has yet to be determined. While the clinical faculty responsible for creating the cases have been enthusiastic and embraced the concept a thorough and complete content analysis of pre- and post-implementation has yet to be conducted. Next steps in the SADI evaluation will include this type of review.

### Opportunities to interact with diverse patients in preclinical years

During the preclinical years, students in the IPM course learn history-taking and clinical exam skills through interactions with standardized patients. As with our patient cases, we believe it is important for the standardized patient population to reflect our community. Barring a few exceptions, most of our standardized patients were white and middle- to upper-class. We wanted to have the opportunity to practice caring for and performing physical exams on other groups of patients we knew we would encounter in our clinical years. Student leaders of the LGBT Interest Group and the LMSA joined together through SADI to advocate for the inclusion of diverse patient populations in the standardized patient setting. Hispanic/Latino and LGBT populations face particular health disparities for various reasons, including physician prejudice, discrimination, lack of training, and language barriers ( Snowdon, 2010; Morales *et al.*, 2015). Understanding these disparities, we combined efforts with IPM teachers to take steps to include interactions with LGBTQ, Hispanic/Latino, and Limited English Proficiency populations in the standardized patient curriculum to provide students practice in caring for these populations.

Teachers and administrators also introduced new lectures and trainings focused on Hispanic/Latino, non-English speaking, and LGBT patients; these innovations ensure students are exposed to these populations early, often, and throughout all four years of medical school. For example, in a new classroom session entitled ‘Introduction to LGBTQ+ Populations’ students are not only provided with written pre-class materials with definitions and helpful communication tools, but also have the opportunity to hear from a trans-woman patient about her personal story and her patient advocacy work. Her journey through the healthcare system to transition has had quite an impact on the students. In one particularly impactful interaction with her primary care provider, when she was looking for help with beginning her transition, she received a response that was much less than compassionate. Her doctor told her, “Well, I promised to do no harm and you know you wouldn’t make a very pretty woman anyway.” Patient panels such as this provide students with more salient examples of the diverse patients they will encounter in clinical practice.

### Extracurricular programs

**Figure 1.**
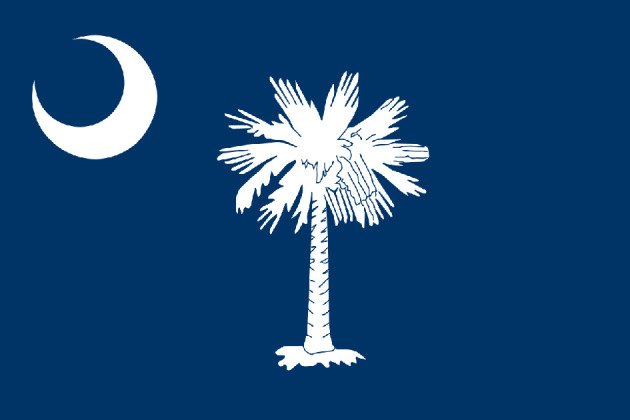
South Carolina State Flag

We recognized another avenue for positive change in our medical school - extracurricular programs to facilitate conversations about diversity and inclusion. Many of has had participated in activities in college or heard from friends at other schools about impactful events they attended, and we wanted a similar platform for discussion and growth at UofSC SOMG,To provide a platform for students to ask difficult questions, we developed an event entitled *Between* *Two Palms*, loosely based on the popular celebrity talk show *Between Two Ferns*; the palms are representative of the state of South Carolina as displayed on the state flag ( Figure 1).

This image of the South Carolina State Flag is available through the Creative Commons CC) 1.0 Universal Public Domain Dedication and can be found at https://commons.wikimedia.org/wiki/File:Flag_of_South_Carolina.svg

We collected anonymous student questions, on topics of adversity, socioeconomic barriers, immigration, religion, race, and gender in medicine. We invited respected physicians, faculty members, and community leaders to serve as guest speakers to answer the students’ questions. *Between Two Palms* debuted at UofSC SOM Greenville in February 2018 as a 90-minute evening event. Student-generated questions included the following: “What characteristic traits/attributes makes a female successful in a surgical specialty?”, “Do you believe affirmative action will always hold a place in professional education? How should medical schools balance the need for diversity with increasingly demanding application requirements?”, and “Is it possible to go your entire career as a doctor without killing someone? What do you do when your mistake leads to someone’s death?” We have since hosted four additional iterations of the program to facilitate deeper discussions, create protected space for dialogue on differing perspectives, and broaden students’ awareness of issues facing medicine today. Other memorable questions from subsequent events included, “While medicine is shifted away from using terms named after Nazis, such as ‘Wegener’s Granulomatosis’, there remain many tools and procedures named after J. Marion Sims, an early gynecologist who experimented on enslaved women. Why do you think this is and do you think we will shift away from using his name as well?”, “What is your practice doing to address the high rate of maternal mortality among African American women?”, and “Do you think abortion should be a right?”.

*Between Two Palms* was created to foster dialogue among students and bring visibility to the diversity within the UofSC SOM Greenville community. We obtained feedback from attendees of the event using audience response technology, by which attendees were invited to submit one- to three-word descriptors in response to the program. Feedback from over 80 students at the first event was overwhelmingly positive, as highlighted by the following descriptors: “provoking”, “honesty”, “worthwhile”, “encouraging”. In all iterations of the program, negative feedback has been exceedingly rare and typically reinforces the need for more open, respectful conversations among students with differing perspectives. Faculty members and physicians who participated as panelists for the events have offered their excitement that these important conversations are happening in medical school now; most also lamented the fact they did not have these opportunities during medical school. We now host *Between Two Palms* bi-annually to continue to provoke thoughtfulness and dialogue about diversity, discrimination, inclusivity, and bias.

## Future Directions

Through SADI, we continue to advocate for diversity and inclusion through increased support for UIM students, inclusive health disparities education, and training in caring for diverse populations. Since its inception, our committee has been further legitimized by official recognition as part of the Student Government Association and the creation of formal student positions, including two peer-elected representatives from each class. These measures ensure consistent interactions between student advocates and faculty and staff and continuity of the committee and its mission for years to come; they also provide opportunities for visibility and representation of UIM students in leadership positions. In 2019, the UofSCSOMG created a new staff position, Director of Multicultural Affairs, and a new faculty position, the Assistant Dean of Admissions. The responsibilities of these positions include recruitment of UIM students, serving as mentors for SADI, and creating programming to support diverse students on campus. We have accomplished so much in the way of the SADI aims due in large part to the support and enthusiasm of the UofSC SOM Greenville faculty, staff, and administration, but also because we have worked together to create infrastructure for continued work in the areas of diversity, equity, and inclusion.

In line with the concept of patient-centered care, SADI believes it imperative to include the perspective of the patient in what innovations we recommend to achieve diversity and inclusion. The conversation about how to better prepare medical students for the actualities of medical practice necessarily involves the voice of the underrepresented and disadvantaged patient. Their perspective allows us insight into the experience of the diverse patients that we will most definitely encounter as physicians. For these reasons, we anticipate recommending a patient serve on the curriculum committee for UofSC SOM Greenville.

Additionally, within our academic health system, we have the Patient Engagement Studio, established with the mission to bring patient perspectives into health research and innovation. This diverse patient panel has been incorporated into some of the initiatives at the medical school; these patients have reviewed student and faculty research studies and assisted in grading student projects for accessibility and readability in the second year. We plan further partnerships with this patient panel to further review innovations within our medical school.

While our successes demonstrate the power of students to innovate and advocate for diversity and inclusion, we continue to promote increased recognition of the importance of these domains at the medical school and within the associated healthcare system. We have included the document that outlines many of the projects and initiatives that SADI hopes to achieve for UofSC SOM Greenville (Appendix1).

We acknowledge the importance of continuous evaluation of our efforts toward diversity and inclusion. While we have not yet engaged in any formal evaluation of the approaches described in this report, we use implementation of initiatives and compliance with changes as measures of success, and we consistently solicit feedback from all stakeholders on areas for improvement in these approaches. In time, we aim to measurably quantify the impact of SADI and its programs on medical students at UofSC SOM Greenville. First on the agenda, we will determine how effective the IPM checklist has been in conveying diversity by comparing cases from years prior to its implementation to cases from years after its implementation.

## Conclusions and Recommendations

Students have the power to design and advance innovations in diversity and inclusion. However, for their efforts to be swift and successful, administration and leadership must be involved, as they are more permanent fixtures of the institution and they have the power to directly change their practices. The formation of a formally recognized student advocacy committee, followed by curricular modifications and extracurricular programs, demonstrates how a ground-up approach may upend a top-down tradition for achieving diversity and inclusivity in the medical education system. Administration and leadership can support student-led initiatives by listening to students, connecting them with the appropriate persons to present their concerns and suggestions to, providing instrumental and informational social support and professional mentorship, and implementing changes in their individual educational practices, as exemplified by the actions of the faculty and staff at UofSC SOM Greenville.

Despite LCME requirements for accreditation, many medical schools still grapple with challenges in the domains of diversity awareness and cultural competence. It is imperative that medical schools fill the gaps in diversity education. It is equally important that medical schools create inclusive places of learning, teaching, and working to attract more diverse applicants and graduate more diverse physicians, representative of the increasingly diverse U.S. population. The early outcomes of our work demonstrate that efforts led by students and embraced by teachers are one way of stimulating necessary change. Future research will investigate the specific impact of our extracurricular programs and curricular changes; although their prompt incorporation is already considered a success, as it represents institutional willingness to adapt and improve to better address physician diversity and mitigate health inequity. Because students and recent graduates hold the unique perspective of lived experience, their voices speak to the areas within the curriculum and the culture that could better prepare them to work with diverse populations; their experiences should guide changes.

Student interest groups and national associations are abundant within medical schools and often reflect the diversity of the student bodies. Unfortunately, these organizations often function as silos. Our experience reiterates a widely recognized phenomenon: that a unified, organized group is more effective than individuals in working toward the same mission. Administration and leadership who encourage a student coalition, with representatives from varied interest groups, may have greater success in implementing institutional initiatives. This phenomenon stands as an important point for medical schools seeking to achieve diversity and inclusion. The SADI model may be especially effective in overcoming barriers faced by student groups at institutions without existing central support, such as a formal Office of Diversity.

Herein we have described the development and implementation of programs, initiatives, and cultural changes at UofSC SOM Greenville, initiated by students and embraced by leaders and administrators. This approach was effective at our medical school because all stakeholders adopted the SADI mission. Together we shared the vision of and worked towards the creation of a more just, equitable, diverse, and inclusive academic curriculum and school culture. We do not believe these results would be reproducible without all parties working together. In order to apply the SADI model elsewhere, faculty, staff, administration, and leadership must provide spaces for conversations about difficult topics and be open to listening to and learning from students. Students must also be prepared to patiently yet persistently suggest specific changes. From our experience, we know that clear communication and open dialogue about areas for improvement, trusting relationships, willing compromises, small successes, and practical proposals ultimately lead to the implementation of useful changes and the innovation of meaningful programs within the medical education system.

## Take Home Messages

• Students have the power to design and advance innovations to achieve diversity and inclusion within the medical education system.
• Faculty and staff involvement in medical student advocacy efforts is more likely to yield successful results.
• Students offer the unique perspective of lived experience; teachers should solicit this perspective to improve the efficacy of their teaching methods.
• Students and teachers of diverse backgrounds and experiences, united by common ideals and goals, can be effective in creating positive institutional change.
• Faculty and staff must create protected spaces for dialogue on difficult topics surrounding diversity and inclusion.

## Notes On Contributors

**Julia L. Moss** is a fourth-year medical student at the University of South Carolina School of Medicine Greenville and a founding member of the Student Advocates for Diversity and Inclusion. ORCID iD: https://orcid.org/0000-0002-5460-2811

**Eliza Hardy** is a fourth-year medical student at the University of South Carolina School of Medicine Greenville and a founding member of the Student Advocates for Diversity and Inclusion.

Student doctor **Keiko Cooley** is a third-year medical student at the University of South Carolina School of Medicine Greenville and a founding member of the Student Advocates for Diversity and Inclusion.

**Samantha Cuffe (Shelhoss)** is a third-year medical student at the University of South Carolina School of Medicine Greenville and a founding member of the Student Advocates for Diversity and Inclusion. ORCID iD: https://orcid.org/0000-0001-9710-3572

**Madeline Lang** is a fourth-year medical student at the University of South Carolina School of Medicine Greenville and a founding member of the Student Advocates for Diversity and Inclusion.

**Ann Blair Kennedy, DrPH** is a Clinical Assistant Professor at University of South Carolina School of Medicine Greenville and the Director of the Patient Engagement Studio for Prisma Health - Upstate Health Science Center. Dr. Kennedy is also a faculty mentor to the Student Advocates for Diversity and Inclusion. ORCID iD: https://orcid.org/0000-0002-3518-6314

## Appendices

### Appendix 1. Student Advocates for Diversity and Inclusion Action Items

*Priority 1* - Support: to contribute to an environment that fosters all students, staff, and faculty

• Establish Office of Diversity and Inclusion
  • Create position of Dean of Diversity and Inclusion
  • Allocate funding for activities/initiatives overseen by the Office
    • Consider consolidating minority interest group budgets
    • Apply for grants, communicate with donors
• Combine efforts with [associate healthcare system]
  • Ensure one representative at each SADI meeting
  • Centralize planning and promotion of diversity events
  • Encourage student attendance at Prisma Health Diversity Events
• Public expression of commitment to diversity and inclusion
  • “Our Vision of Diversity” (UofSC SOM Greenville assessment and plan)
  • Annual Dean’s Hour focusing on Diversity and Inclusion
• Annual diversity report by Dean of Diversity and Inclusion
  • Student, faculty and staff demographics
  • Analysis of grading system including Honors Designations
  • Analysis of awards
  • Activity/Program involvement in arena of Diversity and Inclusion
  • Faculty and student professional development projects
  • Analysis of MedEx as Pipeline for UIM, first-generation, rural, and low-SES students
  • Briefly summarize reports of discrimination and detail how these incidents are resolved and solutions to prevent in future
• Faculty Recruitment
  • Organize professional development of existing faculty
  • Implicit bias training
  • Workshop on recognizing and addressing microaggressions
  • Intentionally recruit diverse physicians as small group mentors for IPM
  • Include statement on diversity on all new faculty employment applications
  • Consider mandating consideration of at least one female and/or minority person for every open faculty position (“Rooney Rule”)
• Admissions Committee: mandate position for fourth-year SADI as voting member
• Early direct intervention and support for board exam preparation for UIMs who are academically at risk
  • Establish criteria for intervention
  • Create algorithm for intervention

*Priority 2* - Curriculum Development: to encourage the integration of diversity and inclusion within the curriculum of medical school student training

• Diversity and Inclusion Module
  • One-hour lecture to be incorporated into first-year orientation week
  • Fostering Belonging Workshop at Northeastern University
• Freshman Summer Reading List
• Standardized Patient Program
  • LGBTQ scenario
  • Low English Proficiency scenario
  • Responding to discrimination/harassment in healthcare scenario
• Language Services Module
  • Provide lecture on interpreter etiquette and accessibility during third-year orientation
  • Require all students to sign Language Services Contract
  • Publish resources for Language Services Proficiency Exam (LPSE)
  • Consider recognition for students who have passed the LSPE
• Create research database for SADI and minority interest groups
  • Project ideas
  • Past publications
  • Presentation opportunities
  • At least one poster annually at university showcase
  • At least one presentation annually at national conference
• Curriculum Committee: add patient advocate from Patient Care Studio as non-voting member; add SADI student as a member
• Health Equity Distinction Track
• Spanish Language Distinction Track

*Priority 3* - Visibility: to support student organizations with a focus on diversity, facilitate meaningful discussion around topics of diversity and inclusion, and increase visibility and inclusion on campus

• Created more aggressive strategy for minority student recruitment by...
  • Expanding and enhancing existing partnerships with historically black colleges and universities
  • Networking and presenting at national undergraduate minority student conferences
• Allocation of funds for Minority Interest Groups
• Diversity Grand Rounds
• Multicultural Graduation Recognition for UIM Students
• Diversity in Medicine Conference, University of Michigan
  • Send newly elected first-year representatives annually
  • Send Dean for Diversity and Inclusion annually
• Docs for Docs (documentary screening/discussion)
• Continue Between Two Palms bi-annually
  • Consider students as guest speakers
