# Supplementary material for: Students Advocating for Diversity in Medical Education
Source: MedEdPublish (2016). 2020 Jan 8;8:159. Originally published 2019 Aug 5. [Version 2] doi: 10.15694/mep.2019.000159.2 (PMC10712464; doi:10.15694/mep.2019.000159.2)
Supplement: Peer review history

# Reviewer report: Reviewer response for version 2

**DOI:** 10.21956/mep.20251.r31418

**Published:** 2020-04-22

**Authors:** Ken Masters

**Affiliations:** 1 Sultan Qaboos University

This review has been migrated. The reviewer awarded 4 stars out of 5 A well-written paper on a difficult and elusive subject, and one that changes progressively. The authors have done well to address the issues raised by the reviewers of the first version, and have produced a worthwhile read.

Reviewer Expertise:

NA

No decision status is available

# Reviewer report: Reviewer response for version 2

**DOI:** 10.21956/mep.20251.r31419

**Published:** 2020-03-05

**Authors:** Trevor Gibbs

**Affiliations:** 1 AMEE

This review has been migrated. The reviewer awarded 4 stars out of 5 Although I did not write a written review the first version of this paper, I did read it and would agree with my two colleagues that the paper would benefit from a more descriptive one. I am pleased to say that the students have written a very good paper here and one that all curriculum developers should read. It is difficult introducing such a complex issue into the curriculum, but the ideas created and described by the authors are very helpful.

Reviewer Expertise:

NA

No decision status is available

# Reviewer report: Reviewer response for version 1

**DOI:** 10.21956/mep.19126.r27614

**Published:** 2019-08-25

**Authors:** Susan Van Schalkwyk

**Affiliations:** 1 Stellenbosch University

This review has been migrated. The reviewer awarded 3 stars out of 5 I too enjoyed reading this paper. Students undoubtedly ‘have the power to design and advance innovations’ in many areas, including in diversity and inclusion, and it is therefore important to document approaches that respond to this thinking. It was refreshing to read about an intervention to effect change that has intentionally incorporated elements that are key for change to occur – breaking down silos, incorporating all role-players, having a structured process with clear milestones, adopting a multi-pronged approach, and so forth. The paper is generally well-written and the authors have avoided emotive language in spite of the potentially emotive nature of the topic. I would agree with the previous reviewer that offering more examples, perhaps sharing some of the student generated questions that have informed the ‘Between two palms’ discussions, would add value. It would also have been useful to know what informed the development of the checklist and how this process occurred. Good luck with your ongoing endeavors in this area.

Reviewer Expertise:

NA

No decision status is available

# Reviewer report: Reviewer response for version 1

**DOI:** 10.21956/mep.19126.r27613

**Published:** 2019-08-11

**Authors:** Judy McKimm

**Affiliations:** 1 Swansea University

This review has been migrated. The reviewer awarded 3 stars out of 5 I really enjoyed this paper and the description of the approach taken by students to develop this engagement and advocacy around diversity and inclusion. You demonstrate a systematic approach to developing from ideas to action. I would have liked to hear more about longer term sustainability and also some case studies and richer description of the issues identified would have brought this more to life, well done though.

Reviewer Expertise:

NA

No decision status is available
